# Supplementary material for: Machine learning quantum phases of matter beyond the fermion sign problem
Source: Sci Rep. 2017 Aug 18;7:8823. doi: 10.1038/s41598-017-09098-0 (PMC5562897; doi:10.1038/s41598-017-09098-0)
Supplement: Supplementary file 1 — Supplementary File [file 41598_2017_9098_MOESM1_ESM.pdf]

Supplementary Material:
Machine learning quantum phases of matter beyond the fermion sign problem

Peter Broecker,[1] Juan Carrasquilla,[2] Roger G. Melko,[2,3] and Simon Trebst[1]

[1]*Institute for Theoretical Physics, University of Cologne, 50937 Cologne, Germany*
[2]*Perimeter Institute for Theoretical Physics, Waterloo, Ontario N2L 2Y5, Canada*
[3]*Department of Physics and Astronomy, University of Waterloo, Ontario, N2L 3G1, Canada*

(Dated: July 14, 2017)

I. HUBBARD STRATONOVICH TRANSFORMATION IN THE MAGNETIC CHANNEL

Here we present additional information on the use of the auxiliary fields as the input to the neural network. We consider again the spinful Hubbard model on the honeycomb lattice and this time choose a Hubbard-Stratonovich transformation that couples to the magnetization instead of the charge as done earlier. It thereby breaks SU(2) symmetry for each of the auxiliary field configurations which is, however, restored after summation over all auxiliary field configurations. The same neural network architecture used previously is employed here and trained with the same number of configurations at two extremal points of the on-site interaction U. The resulting prediction for the intermediate U-values is depicted in Fig. 1.

Looking at the two side panels, one clearly sees that in the case of strong coupling, distinct lines appear along the direction of projection time in contrast to the seemingly random configuration at weak coupling strength. With the same number of samples and training runs, the network is able to distinguish these two phases to good but not perfect accuracy as indicated by the fact that samples drawn from the training points, highlighted with red dots, are not exclusively associated with their respective phases. More strikingly, the critical interaction U_c is found at around $U_c = 8.0$, far away from the actual point of transition. We would like to emphasize that the result might be improved by choosing a different layout of the neural network or different convolutional filters that, for example, take into account the spatial arrangement of the sites. Our intent in this study, however, was to devise a robust method that requires as little fine-tuning of the neural network as possible but still provides us with accurate results. The Green's functions therefore appear to be better suited for achieving this goal.

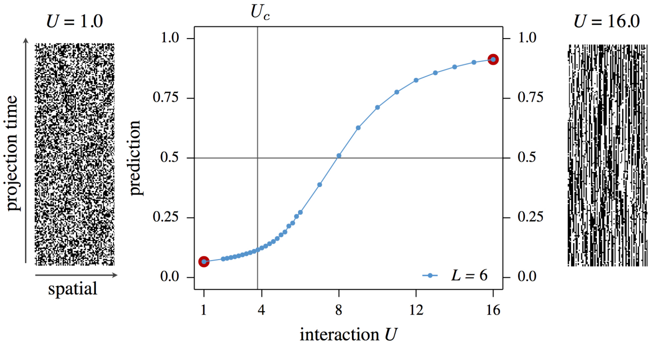

Figure 1. (Color online) Results from training the neural network on auxiliary field configurations of a spinful Hubbard model on a $2 \cdot 6 \times 6$ lattice with on-site interaction U using a Hubbard-Stratonovich decoupling in the magnetic channel. Reference points for training were $U = 1.0$ and $U = 16.0$, marked by red dots in the figure. The neural network is able to distinguish the two phases, but the prediction of the critical point is not very accurate.
